# Phenotype and genotype analyses of Chinese patients with autosomal dominant mental retardation type 5 caused by *SYNGAP1* gene mutations

**DOI:** 10.3389/fgene.2022.957915

**Published:** 2022-12-13

**Authors:** Yanxin Wang, Yuqiang Lv, Zilong Li, Min Gao, Xiaomeng Yang, Yue Li, Jianguo Shi, Zaifen Gao, Yi Liu, Zhongtao Gai

**Affiliations:** ^1^ Department of Pediatrics, Children’s Hospital Affiliated to Shandong University, Ji’nan, China; ^2^ Pediatric Research Institute, Children’s Hospital Affiliated to Shandong University, Ji’nan, China; ^3^ Shandong Provincial Clinical Research Center for Children’s Health and Disease, Ji’nan, China; ^4^ Epilepsy Center, Children’s Hospital Affiliated to Shandong University, Ji’nan, China

**Keywords:** autosomal dominant mental retardation type 5, mutation, *SYNGAP1*, intellectual disability, developmental delay, next-generation sequencing, epilepsy

## Abstract

**Background:** Autosomal dominant mental retardation type 5 (MRD5), a rare neurodevelopmental disorder (NDD) characterized by intellectual disability (ID), developmental delay (DD), and epilepsy predominantly, is caused by a heterozygous mutation in the *SYNGAP1* gene. *SYNGAP1* mutations have been rarely reported in the Chinese population. Here, we present an investigation of *SYNGAP1* mutations in a clinical cohort with ID and DD in Shandong, a northern province in China, to further explore the genotype and phenotype correlations.

**Methods:** A retrospective study was conducted on 10 children with *SYNGAP1* mutations presenting ID, DD, and epilepsy who were diagnosed between January 2014 and May 2022. Clinical data and genetic tests were collected. Treatment and regular follow-ups were carried out to pay close attention to the prognosis of the patients.

**Results:** We described 10 unrelated affected individuals with *SYNGAP1* mutations, displaying ID, DD, epilepsy, or seizures. All mutations of *SYNGAP1* in the 10 patients were *de novo*, except patient 3 whose father was unavailable, including five nonsense mutations, two frameshift mutations, two splicing mutations, and one codon deletion. Among these mutations, five were novel and the other five were previously reported. Significantly, all patients with epilepsy were sensitive to anti-seizure drugs, especially sodium valproate. Furthermore, rehabilitation training seemed to exert a more improved effect on motor development than language development for the patients.

**Conclusion** The 10 patients carrying *SYNGAP1* mutations were diagnosed as MRD5. Five novel genetic mutations were found, which expanded the mutational spectrum of the *SYNGAP1* gene. The identification of these mutations in this study helps explore the relationship between genotypes and phenotypes and contributes to genetic counseling and therapeutic intervention for patients with MRD5.

## Introduction

Autosomal dominant mental retardation type 5 (MRD5; OMIM #612621), a recently described single-gene disorder, is defined as a rare neurodevelopmental disorder characterized by moderate to severe intellectual disability (ID) and psychomotor developmental delay (DD) in the first year of life, with a high frequency of comorbid epilepsy and autism spectrum disorder (ASD) (Deciphering Developmental Disorders Study, 2015; Agarwal et al., 2019; Verma et al., 2020). With the rapid development of molecular diagnostic techniques, the heterozygous loss-of-function mutations of the synaptic Ras GTPase-activating protein 1 (*SYNGAP1*) gene (OMIM #603384) have been deemed the genetic etiology of MRD5 in an autosomal dominant manner (Parker et al., 2015; Weldon et al., 2018). Reportedly, the estimated incidence of *SYNGAP1* mutations accounts for ∼0.75% of patients with neurodevelopmental disorders (NDDs) (Berryer et al., 2013; Deciphering Developmental Disorders Study, 2015; Mignot et al., 2016; Weldon et al., 2018; Zhang et al., 2021). As merely more than 200 cases of MDR5 are reported worldwide, the definite incidence has not been established to date (Gamache et al., 2020; Zhang H et al., 2020).

The *SYNGAP1* gene is on chromosome 6p21.3 and consists of 19 exons encoding synaptic RAS-GTPase-activating protein (SynGAP), which is localized to dendritic spines in the postsynaptic density (PSD) of excitatory glutamatergic neurons and primarily expressed in the developing brain, particularly the forebrain and the hippocampus. By alternative splicing, SynGAP could produce distinct functional protein isoforms and perform different functions, such as regulation of neural excitability, development of dendritic arborization, maturation of the dendritic spine, and plasticity of synapses (Araki et al., 2015; Meili et al., 2021; Kilinc et al., 2022). There are various predicted functional domains in SynGAP, including the pleckstrin homology (PH) domain (pos. 150–251) in the N-terminus; C2 domain (pos. 263–362) and RasGAP domain (pos. 392–729) in the core region; and the SH3 domain (pos. 785–815), the coiled-coil (CC) domain (pos. 1,189–1,262), and other C-terminal domains that resulted from alternative splicing such as QTRV for isoform α1, TADH for isoform α2, PRGH for isoform *β*, and LLIR for isoform *γ*, although their exact functions and molecular mechanisms remain unclear (Berryer et al., 2013; Gamache et al., 2020; Kilinc et al., 2022). Loss-of-function mutations in *SYNGAP1* resulting in SynGAP haploinsufficiency contribute to a constellation of symptoms of ID, DD, and seizures, which have recently been termed a neurodevelopmental disorder—MRD5 (Agarwal et al., 2019; Kilinc et al., 2022).

To date, reported cases regarding MRD5 have been predominantly from Europe, whereas only a few cases have been elucidated previously in China (Pei et al., 2018; Lu et al., 2019; Gao et al., 2020; Zhang et al., 2021). In the study, we present the clinical and mutational characteristics of additional 10 Chinese patients with MRD5 identified by next-generation sequencing (NGS) from a clinical cohort of 1,986 cases with NDDs manifesting ID, DD, epilepsy, and ASD between January 2014 and May 2022 in Shandong Province of China, and five novel mutations in the *SYNGAP1* gene were discovered.

## Materials and methods

### Patients

Five male and five female patients from unrelated families were identified and diagnosed as having MRD5 from a clinical cohort of 1,986 cases with NDDs between January 2014 and May 2022 at Children’s Hospital affiliated to Shandong University. All patients from the Han Chinese population in Shandong Province in China were examined and clinically diagnosed by experienced pediatric neurologists of the hospital according to the DMS-5 criteria. All clinical data were gathered, including family history, clinical features, laboratory tests, magnetic resonance imaging (MRI) of the brain, and electroencephalogram (EEG).

### Exome sequencing

Peripheral blood samples were obtained from all patients and their parents except patient 3, whose father was unavailable. Genomic DNA was extracted from blood samples with the TIANamp Blood DNA Kit (Tiangen, China) in accordance with the standard protocol. The whole exome sequencing (WES) with the SeqCap EZ Choice XL Library (Roche NimbleGen) was used to capture the exon regions and adjacent intron regions (50 bp) (Illumina, America). The mean sequencing depth of the targeted areas was 134.09X, of which 97.71% of the target sequences were more than 20X.

Sequencing data were compared with the UCSC hg19 human reference genome sequence using NextGene V2.3.4 software to identify genetic mutations. At the same time, mutations were further annotated by NextGene V2.3.4 and associated with multiple databases, such as 1000 Genomes, ExAC, dbSNP, HGMD, ClinVar, ESP6500, OMIM, and Inhouse databases. Then, pathogenicity was predicted by PolyPhen-2, SIFT, MutationTaster, and REVEL. When all the aforementioned analyses were finished, the obtained mutations eventually were the candidates for pathogenic mutations. Thereafter, the pathogenicity assessment of genetic mutations was carried out, which was based on the 2015 American College of Medical Genetics and Genomics (ACMG) guidelines (Richards et al., 2015).

### Validation of genetic mutations

Sanger sequencing was applied to validate the mutations detected by exome sequencing in the patients and their parents. Primer sets were designed by Primer Premier v5.0 software. Furthermore, AmpliTaq Gold^®^ 360 DNA Polymerase (Applied Biosystems) was used for PCR amplification. PCR products were then purified and sequenced on an ABI Prism 3700 automated sequencer (Applied Biosystems, CA, United States).

### Statistical analysis

Statistical analysis was performed with SPSS 19.0 software. Chi-squared tests were used to test the significance of mutations in different groups, and results were regarded as statistically significant when the *p*-value was <0.05.

## Results

### Clinical findings

Five male and five female children were recruited into our study with a mean diagnostic age of 41.6 months (ranging from 14 months to 89 months). Intellectual disability and global developmental delay of varying levels were observed in all the patients, of whom five (P4, P5, P6, P7, and P10) had seizures, four (P2, P4, P9, and P10) showed ataxia or gait abnormalities, and six (P2, P4, P5, P6, P9, and P10) were observed to have behavioral problems, while one patient (P5) was clinically diagnosed with ASD. In addition, one patient P3 manifested hypotonia of four limbs, especially the upper ones, and one patient P9 had internal strabismus of the eyes. All the patients had impaired speaking ability, particularly three patients P4, P6, and P7 who remain verbally disabled.

The age of seizure onset for the five affected individuals (P4, P5, P6, P7, and P10) varied from 18 months to 60 months (mean age 37.8 months). The types of seizures monitored by EEGs consisted of atypical absence (4/5), eyelid myoclonia (1/5), atypical absence with myoclonus (1/5), myoclonic seizure (1/5), atonic seizure (1/5), myoclonus with atonic seizure (1/5), and unclassified fall attack (1/5). Additionally, the EEG of a patient (P3) was abnormal with multifocal waves and spikes without a predominant rhythm, while seizure onset was not observed. As for the five patients with seizures, generalized seizures with diffuse slow wave backgrounds occurred in four individuals (P4, P6, P7, and P10), but the focal seizure was only noticed in one patient P5, and no trigger of seizures was identified.

Furthermore, a normal or non-specific presentation was observed in brain MRI, while patient 3 displayed a slightly widened extracerebral lacuna in the left temporal pole and two patients (P8 and P9) showed the possibility of the terminal band in the bodies of the lateral ventricles. Additionally, no abnormality in karyotypes, metabolic screening, and the function of the thyroid glands was observed. However, the levels of growth hormone were obviously below normal, which remained to be explored further. Clinical data are shown in detail in Table 1.

**TABLE 1:** Clinical information of 10 Chinese patients.

Clinical information	P1	P2	P3	P4	P5	P6	P7	P8	P9	P10
Gender	Female	Female	Male	Male	Female	Male	Male	Female	Female	Male
Age	30 m	89 m	14 m	58 m	31 m	24 m	30 m	34 m	43 m	63 m
ID	+	+	+	+	+	+	+	+	+	+
IQ	−	44	N/A	43	−	−	−	−	43	−
Language delay	+	+	+	+	+	+	+	+	+	+
Age of speaking	N/A	N/A	N/A	−	N/A	−	−	18 m	N/A	N/A
Current speech ability	Two–three words	Simple sentences	Two–three words	Verbal disability	Two words	Verbal disability	Verbal disability	Several words	Four words	A few words
Motor delay	+	+	+	+	+	+	+	+	+	+
Age of walking	N/A	16 m	Unable to walk	18 m	18 m	20 m	27 m	18 m	N/A	N/A
Impairment of motor	Mild	Moderate	Severe	Moderate	Moderate	Moderate	Moderate	Mild	Moderate	Severe
Hypomyotonia	−	−	+	−	−	−	−	−	−	−
Ataxia	−	+	N/A	+	−	−	−	−	+	+
ASD	−	−	−	−	+	−	−	−	-	−
Behavioral problems	−	+	N/A	+	+	+	−	−	+	+
Malformations	−	−	−	−	−	−	−	−	Internal strabismus	−
Seizures	−	−	−	+	+	+	+	−	−	+
Age of seizure onset	−	−	−	55 m	27 m	18 m	29 m	−	−	60 m
Onset type of seizures	−	−	−	FA	AAb	AAb with or without M; At; M; M with At	AAb	−	−	EMA; AAb
Effect of ASMs	−	−	−	Sensitive	Sensitive	Sensitive	Sensitive	−	−	Sensitive
EEG	N/A	N/A	Abnormal	Abnormal	Abnormal	Abnormal	Abnormal	N/A	N/A	Abnormal
MRI	N/A	Normal	Nonspecific	N/A	Normal	Normal	Normal	Nonspecific	Nonspecific	Normal
Karyotype	46, XX	46, XX	46, XY	46, XY	46, XX	46, XY	46, XY	46, XX	46, XX	46, XY
Metabolic screening	N/A	N/A	N/A	Normal	Normal	Normal	N/A	N/A	Normal	N/A
Functions of the thyroid gland	Normal	Normal	N/A	Normal	Normal	N/A	N/A	N/A	N/A	N/A
Growth hormone (≧10 ug/L)	0.23	N/A	N/A	7.73	3.92	N/A	N/A	N/A	N/A	N/A

+, present; −, absent; N/A, not available; y, year(s); m, month(s); FA, fall attack; AAb, atypical absence; M, myoclonia; At, atonia; EMA, eyelid myoclonia; ASM, anti-seizure medication.

### Genetic findings and analysis of novel mutations

**FIGURE 1:**
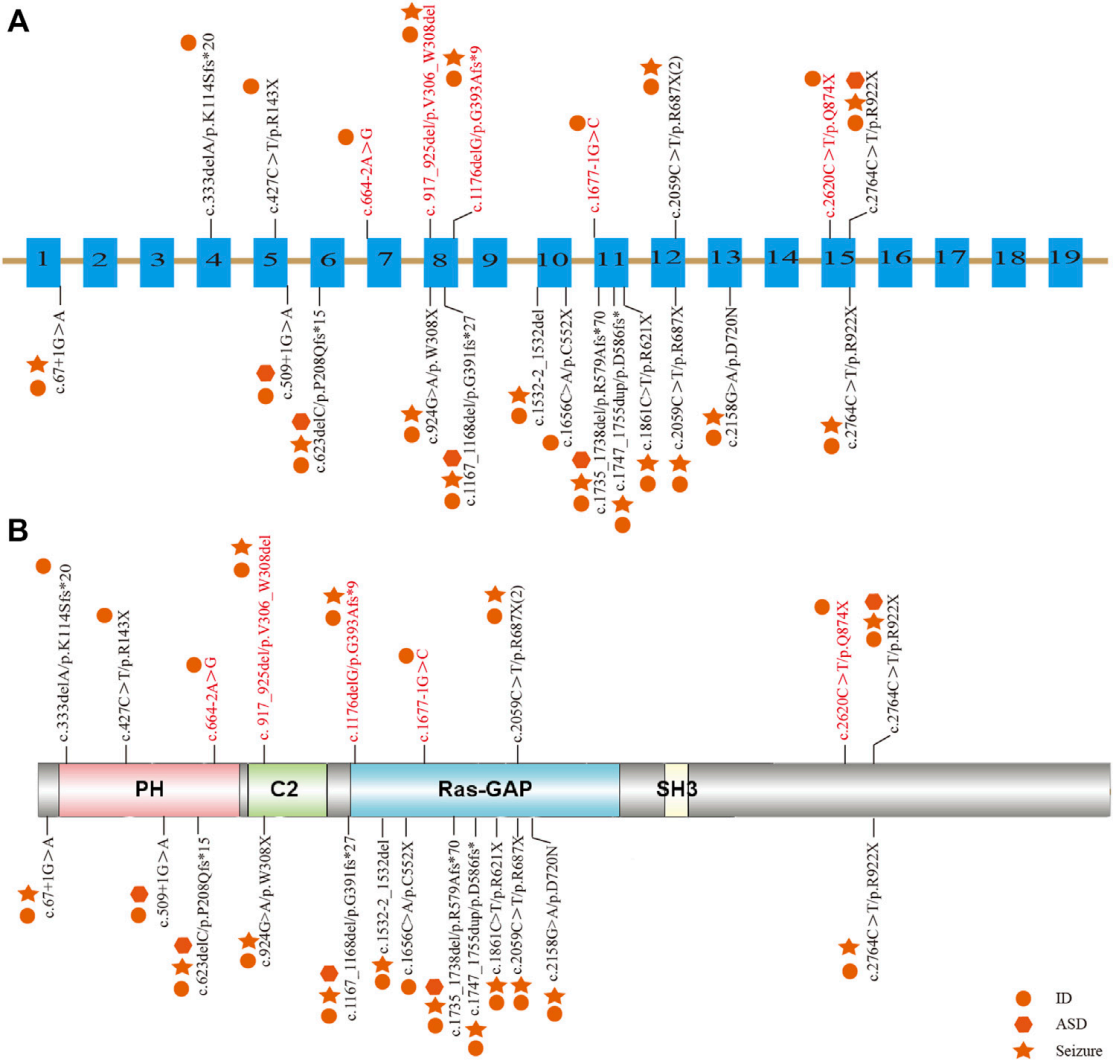
Distribution of mutations in Chinese patients among different exons and domains. The mutations in our study are listed prior to the exons or domains, in which five novel mutations are marked in red. The other mutations from previous studies in China are listed beneath the exons or domains. The various shapes represent different phenotypes, in which the circle means ID/DD, the star means seizure, and the hexagon means ASD. **(A)** Distribution of mutations in the exons of the SYNGAP1 gene. **(B)** Distribution of mutations in the domains of SynGAP.

Among the 10 individuals, nine distinctive mutations were identified; two patients (P7 and P10) shared the same mutation of c.2059C>T (p.R687X), five mutations were novel, and the remaining five were reported (Table 2). All mutations were validated as *de novo* with the exception of patient 3 as his father was unavailable. The 10 mutations consisted of nonsense (5, 50%), frameshift (2, 20%), splicing (2, 20%), and codon deletion (1, 10%). The mutation sites were distributed in exons 4, 5, 8, 12, and 15 and introns 6 and 10 (Figure 1A) and were located on the domains of SynGAP from PH to RasGAP domains, with two on the PH domain, one on the C2 domain, three on the RasGAP domain, and two on the C-terminal unknown domain (Figure 1B). According to the American College of Medical Genetics and Genomics (ACMG) guidelines, eight mutations in nine patients were determined as pathogenic, and one mutation (c.917_925del/p.V306_W308del) was likely pathogenic (Table 2). The five novel mutations, including two splicing, one codon deletion, one frameshift, and one nonsense, were predicted to disrupt the function of the protein.

**TABLE 2:** Genetic information of 10 patients in our study.

Patient	Mutation site	cDNA change	Mutation type	Amino acid change	Source of mutation	Status	ACMG	Pathogenicity
P1	Exon 4	c.333delA	Frameshift	p.K114Sfs*20	*De novo*	Reported in Carvill et al. (2013)	PVS1+PS1+PS2	Pathogenic
P2	Intron 6	c.664–2A>G	Splicing	—	*De novo*	Novel	PVS1+PS2+PM2	Pathogenic
P3	Intron 10	c.1677-1G>C	Splicing	—	Father: not available. Mother: no mutation	Novel	PVS1+PM2+PP3	Pathogenic
P4	Exon 8	c.917_925del	Codon deletion	p.V306_W308del	*De novo*	Novel	PS2+PM2	Likely pathogenic
P5	Exon 15	c.2764C>T	Nonsense	p.R922X	*De novo*	Reported in Parker et al. (2015)	PVS + PS1+PS2+PM2	Pathogenic
P6	Exon 8	c.1176delG	Frameshift	p.G393Afs*9	*De novo*	Novel	PVS + PS2+PM2	Pathogenic
P7	Exon 12	c.2059C>T	Nonsense	p.R687X	*De novo*	Reported in Gao et al. (2018)	PVS + PS2+PM2	Pathogenic
P8	Exon 5	c.427C>T	Nonsense	p.R143X	*De novo*	Reported in Carvill et al. (2013)	PVS1+PS2+PS4+PM2	Pathogenic
P9	Exon 15	c.2620C>T	Nonsense	p.Q874X	*De novo*	Novel	PVS1+PS2+PM2	Pathogenic
P10	Exon 12	c.2059C>T	Nonsense	p.R687X	*De novo*	Reported in Gao et al. (2018)	PVS1+PS2+PM2	Pathogenic

All variants were described by NM_006772.2 for *SYNGAP1* transcript reference sequences using the version of GRCh37/hg19.

### Treatment and medical follow-up

During the mean follow-up period of 33.2 months, all affected individuals were subjected to rehabilitation training for more than one year. Preliminary improvement in motor development could be seen with varying degrees. Almost all patients except patient 3 were equipped with the ability to walk unaided despite the existence of an unsteady gait in three children (P5, P9, and P10). Patient 3 was able to sit unaided for approximately 30 min but had difficulty in standing independently, even though his electromyogram was normal. In contrast to motor development, there was no significant improvement in the language development of the patients, particularly three patients (P4, P6, and P7) who still had verbal disability, whereas the other seven children could only speak few words.

Anti-seizure drugs sodium valproate and lamotrigine were administered to the five patients with epilepsy, demonstrating significant improvement. Patient 4 initially did not take medications under medical supervision until the identical type of seizure occurred once. After sodium valproate was prescribed, seizure was under control eventually. Up to now, patient 4 has been free from seizures for 11 months. Patient 5 was prescribed a medication combination of sodium valproate and lamotrigine and remained seizure-free for 1 year. Patient 6 was administered with sodium valproate and has been seizure-free for more than 3 years. Similarly, patient 7 was also sensitive to valproate, although his EEGs are still abnormal. Sodium valproate also worked for patient 10 but was discontinued for a month, during which the patient experienced seizure recurrence twice. Until sodium valproate was taken again, his seizure was controlled, and this stable status has lasted for 11 months.

### Genotype–phenotype correlation

To elucidate the genotype–phenotype correlation of Chinese patients, we searched all publications regarding *SYNGAP1* mutations in the Chinese population in PubMed and all available Chinese databases, such as Wanfang and China National Knowledge Infrastructure. Thus, data on a total of 23 patients with *SYNGAP1* mutations were summarized, and the patients were divided into four different groups according to their mutation type (nonsense, splicing, frameshift, and missense) in the *SYNGAP1* gene (Pei et al., 2018; Lu et al., 2019; Gao et al., 2020; Zhang et al., 2021). All 23 patients presented ID and DD, of which only three patients with nonsense, frameshift, and splicing mutations were diagnosed as ASD, while the numbers of patients with seizures varied in the four groups. Among them, there were seven (7/10, 70%) patients with seizures in the nonsense group, six (6/7, 85.7%) in the frameshift group, two (2/5, 40%) in the splicing group, and one (1/1, 100%) in the missense group. Statistically, the results exhibited no significant difference among the four groups (Table 3). In terms of the correlation between the phenotypes and the locations of the mutations in the *SYNGAP1* gene, our patients with mutations in exons 4 and 5 displayed milder developmental delay and no comorbid epilepsy, whereas those with mutations in exons 8–15 had moderate to severe clinical manifestations (Figure 1A). However, there was no association between the various manifestations and different mutation domains (Figure 1B).

**TABLE 3:** Comparison of cardinal features in 23 Chinese patients.

	Nonsense	Frameshift	Splicing	Missense
Number	10	7	5	1
Male/female	4/6	5/2	4/1	0/1
Mean age	45.5 m	57.6 m	50.2 m	9 m
ID	10	7	5	1
DD	10	7	5	1
ASD	1	1	1	0
Seizure	7	6	2	1

m, month(s).

## Discussion

The genetic basis of MRD5 has been attributed to the loss-of-function mutation of the *SYNGAP1* gene, which is ranked the fourth most common NDD-related gene among a large spectrum of genes sharing overlapping phenotypes, accounting for ∼0.75% of all NDD patients (Berryer et al., 2013; Deciphering Developmental Disorders Study, 2015; Kilinc et al., 2022). To date, more than 200 patients with *SYNGAP1* mutations associated with various phenotypes, such as ID, DD, epilepsy, ASD, and schizophrenia, have been reported in the Human Gene Mutation Database (HGMD) (Jimenez-Gomez et al., 2019; Gamache et al., 2020; Zhang H et al., 2020). Nevertheless, there has been no established diagnostic criterion for MRD5 until now. Here, we additionally identified 10 affected individuals harboring heterozygous mutations of the *SYNGAP1* gene (five novel and five reported) to further clarify the clinical and genetic characteristics.

The 10 patients in this study were identified from a clinical cohort of 1,986 cases with NDDs. Thus, the incidence of *SYNGAP1* mutation is approximately 0.5% in our NDD cohort, which is a little less than the previous reports (Berryer et al., 2013; Kilinc et al., 2022). The main phenotypes of the 10 patients include ID (10/10, 100%), DD (10/10, 100%), epilepsy (5/10, 50%), and ASD (1/10, 10%). Consistent with previous literature, all patients manifested DD and ID during their first year of life, while impaired global development was the most remarkable feature in their early stages, of which the majority were moderately to severely affected. Compared with motor retardation, language development was impaired more badly with three individuals (P4, P6, and P7) who still appeared speechless in the last follow-up visit (Agarwal et al., 2019; Jimenez-Gomez et al., 2019; Zhang et al., 2021). In addition, the dynamic detection of GH and assessments of physical development also deserved more attention, which had not been reported in the previous literature. In terms of epilepsy, only five (50%) patients in the study were diagnosed, but they were all sensitive to anti-epilepsy drugs, especially sodium valproate, which is different from the previous Chinese study (Zhang et al., 2021). However, the pharmacoresistant rate among all Chinese patients was similar to that of reported foreign patients (Mignot et al., 2016; Agarwal et al., 2019).

Significantly, rehabilitation training exerted a more positive effect on motor development than language development, which is consistent with the previous report (Zhang et al., 2021), but long-term prognosis could not be predicted due to the short follow-up time window, demonstrating that a standardized lifelong follow-up will be needed. In contrast to most studies regarding much younger patients, Prchalova et al. (2017) described an adult female patient with similar phenotypes as other patients carrying a *de novo* splicing mutation (c.1676 + 5G>A) in the *SYNGAP1* gene. Retrospecting her medical history, they noticed that a few of the phenotypes such as growth delay, microcephaly, and seizures could be alleviated or well-controlled with age, whereas several phenotypes such as facial features, unsteady gait, and cognitive and language performance showed a tendency to deteriorate, indicating a specific dynamic of the *SYNGAP1*-related phenotypes from a young age to adulthood. Whether early training and drug interventions could exert an underlying influence on disorder prognosis remains to be observed in the long-term clinical follow-up.

The *SYNGAP1* gene is rather complex and can be expressed in multiple isoforms with different distributions and regulatory functions. Numerous isoforms including three N-terminal (A, B, and C) and four C-terminal (α1, α2, *β*, and *γ*) variations were generated using distinct promoters and alternative splicing, each of which performed its own functions in a distinct subcellular location (Agarwal et al., 2019; Araki et al., 2020; Kilinc et al., 2022). Reportedly, α1 and *β* isoforms are highly enriched in forebrain neurons; while the former mainly focuses on excitatory synapses, the latter spreads over both excitatory and inhibitory synapses (Gou et al., 2020; Araki et al., 2020; Kilinc et al., 2022). Additionally, both SynGAP α1 and α2, two C-terminal isomers , also present opposing effects in the regulation of synaptic strength under the underlying control of the N-terminal sequence (McMahon et al., 2012). However, the unique role of each one or the association among numerous isoforms has not been completely understood. To our knowledge, reported mutations in the *SYNGAP1* gene were involved in different domains from PH, RasGAP to C-terminal unknown domains, but no clear relationship associated with phenotypes was found. Genetically, the priority of mutation types was nonsense, frameshift, splicing, and codon deletion, in turn, in which nonsense mutations were the most common. In our study, five novel mutations were identified, including two splicing mutations (c.664–2A>G and c.1677-1G>C), one frameshift mutation (c.1176delG), one codon deletion (c.917_925del), and one nonsense mutation (c.2620C>T) in five patients, respectively. Nonsense and frameshift mutations are also known as truncated mutations, which are related to truncated proteins and loss of functions directly. The two splicing mutations that occurred in introns disrupt the acceptor sites and, thus, result in errors during the splicing process, causing alterations of the open reading frame and the protein (Anna and Monika, 2018). Codon deletion with deletion of nine bases is an in-flame mutation that could shorten the length of the DNA strand and thus change the structure of the protein, which further results in conformational defects. As a result, the underlying effect of the 10 *de novo* heterozygous mutations is associated with *SYNGAP1* haploinsufficiency, further declaring that the *SYNGAP1* gene indeed acts as a significant component in neurodevelopment. In addition, SynGAP consists of various domains (Figure 1B): the PH domain refers to membrane localization of proteins; the C2 domain is involved in Ca^2+^ binding; the RasGAP domain contains many phosphorylation sites for kinases such as calmodulin-dependent protein kinase II (CaMKII) and participates in the activation of downstream molecules such as Ras and Rap; the SH3 domain mediates protein–protein interactions such as between NMDAR and PSD-95; and the coiled-coil domain is responsible for the multimerization among SynGAP isoforms (Agarwal et al., 2019; Gamache et al., 2020). All mutations in our study are involved in different domains, mainly from PH to RasGAP, and are considered deleterious for damaging the corresponding functional domains of proteins.

Together with 13 other Chinese patients reported previously, no remarkable alteration in mutation types and pathogenicity was observed. Meanwhile, those patients also shared a spectrum of clinical features. With respect to seizure types, eyelid myoclonia and absence appeared to be the most frequent ones. The existence of differences between our retrospective studies and previous reports (Pei et al., 2018; Lu et al., 2019; Gao et al., 2020; Zhang et al., 2021) verified the necessity of a large multicenter prospective cohort study. Additionally, six abnormal EEGs were observed without any seizure onset, and whether anti-seizure medication should be prescribed for them is still unknown due to the absence of established guidelines regarding medication regime and prognosis management at present. Moreover, we analyzed the clinical and genetic features among different mutation types in Chinese patients, but no clue regarding the phenotype–genotype correlation was identified. Vlaskamp et al. (2019) identified the correlation between the phenotypes and mutation locations; that is, milder phenotypes were often relevant to mutations in exons 1–4, whereas more severe phenotypes frequently occurred in exons 8–15. In line with the aforementioned findings, our patients with mutations in exons 4 and 5 displayed milder developmental delay and no comorbid epilepsy, whereas those with mutations in exons 8–15 had moderate to severe clinical manifestations. The exact pathogenic mechanisms have not been fully understood and remain to be explored further.

SynGAP has been considered to play an important role in synaptic plasticity and the development of neurons. The gross structure of an intact synapse consists of a presynaptic membrane, postsynaptic membrane, and synaptic cleft, facilitating information transmission from biochemical neurotransmitters to electrophysiological signals and further activating downstream signaling cascades of the NMDAR-mediated Ras signaling pathways (Araki et al., 2020; Meili et al., 2021). In this pathway, NMDAR is activated by glutamate neurotransmitters released from the presynaptic membrane, resulting in a Ca^2+^ influx into the postsynaptic neuron. Subsequently, calmodulin-dependent protein kinase II (CaMKII) is triggered, and then SynGAP is phosphorylated, further negatively regulating Ras-mediated ERK/MAPK signaling cascades and restraining AMPAR insertion at the postsynaptic membrane (Jeyabalan and Clement, 2016; Meili et al., 2021). Structurally, SynGAP competes for binding to the PDZ domain of scaffolding protein PSD-95 with transmembrane AMPAR regulatory proteins (TARPs), which also facilitates AMPAR trafficking to PSD (Walkup et al., 2016). Thus, *SYNGAP1* haploinsufficiency results in decreased enzymatic activity on Ras and number of binding sites for PSD-95, which further leads to increased AMPAR levels at baseline (Gamache et al., 2020). Aberrant alterations greatly affect neural development, including increased excitability, excitation/inhibition imbalance, and loss of experience-dependent plasticity, which are responsible for cognition, learning, memory, and motor defects (Aceti et al., 2015; Uzunova et al., 2016; Zhang Y et al., 2020; Ni and Li, 2021). In addition, the Ras substrate and other members in the small GTPase-activating protein (GAP) superfamily, such as Rap and Rac, are also regulated by *SYNGAP1*. However, the ability to regulate Rap is even more powerful than that of Ras, although the relevant mechanisms are rarely involved (Agarwal et al., 2019).

Pathogenic processes have not been fully understood on the basis of molecular studies. Thus, animal models are generated for further exploration. Given that *SYNGAP1* is highly conserved across species (Kilinc et al., 2018), animal models provide us with unique insights into the pathogenesis of genetic haploinsufficiency. To date, *SYNGAP1* ^−/+^ mice have been extensively utilized, whereas homozygous *SYNGAP1* knockout mice fail to survive for more than a week, indicating the vital role of SynGAP in postnatal development (Berryer et al., 2013). Using mouse models, Aceti et al. (2015) showed that the deletion of SynGAP exerted a negative effect on the critical development period of cortical synaptogenesis after birth and accelerated dendrite elongation and spine morphogenesis in cortical pyramidal neurons, thus hampering synaptic plasticity relevant to the reorganization of brain circuits depending on experience. With alternative detection of structural abnormalities in the neurons of mutant mice, some progress has been achieved from behavioral studies of mouse models. In line with previous reports (Ozkan et al., 2014; Berryer et al., 2016), Nakajima et al. found that heterozygous knockout mice exhibit increased locomotor activity, decreased prepulse inhibition, and impaired working and reference spatial memory, which was determined by a series of comprehensive behavioral tests (Nakajima et al., 2019). These findings recapitulate phenotypes in patients with *SYNGAP1* mutations and can be combined with phenotypes at the molecular level, realizing the effect of mutual complementation.

## Conclusion

Collectively, our study demonstrated the clinical characteristics of 10 Chinese individuals with MRD5 caused by *SYNGAP1* mutations, and half of these mutations were novel, which contributed to expanding the mutational spectrum of the *SYNGAP1* gene. Furthermore, nonsense mutations remained the most frequent mutation type. The identification of these mutations in this study is helpful in exploring the relationship between genotypes and phenotypes. More importantly, it is likely that several underlying phenotypes have not yet emerged because all the probands are in the early stage of growth and development, implying that a long-term follow-up is essential.

## Data Availability

The data presented in this study are deposited in the GenBank repository (https://www.ncbi.nlm.nih.gov/Genbank), accession number ON996974 to ON996976, and OP161168 to OP161171.
