# Supplementary figures and images for: Phenotype and genotype analyses of Chinese patients with autosomal dominant mental retardation type 5 caused by SYNGAP1 gene mutations
Source: Front Genet. 2022 Dec 13;13:957915. doi: 10.3389/fgene.2022.957915 (PMC9792850; doi:10.3389/fgene.2022.957915)

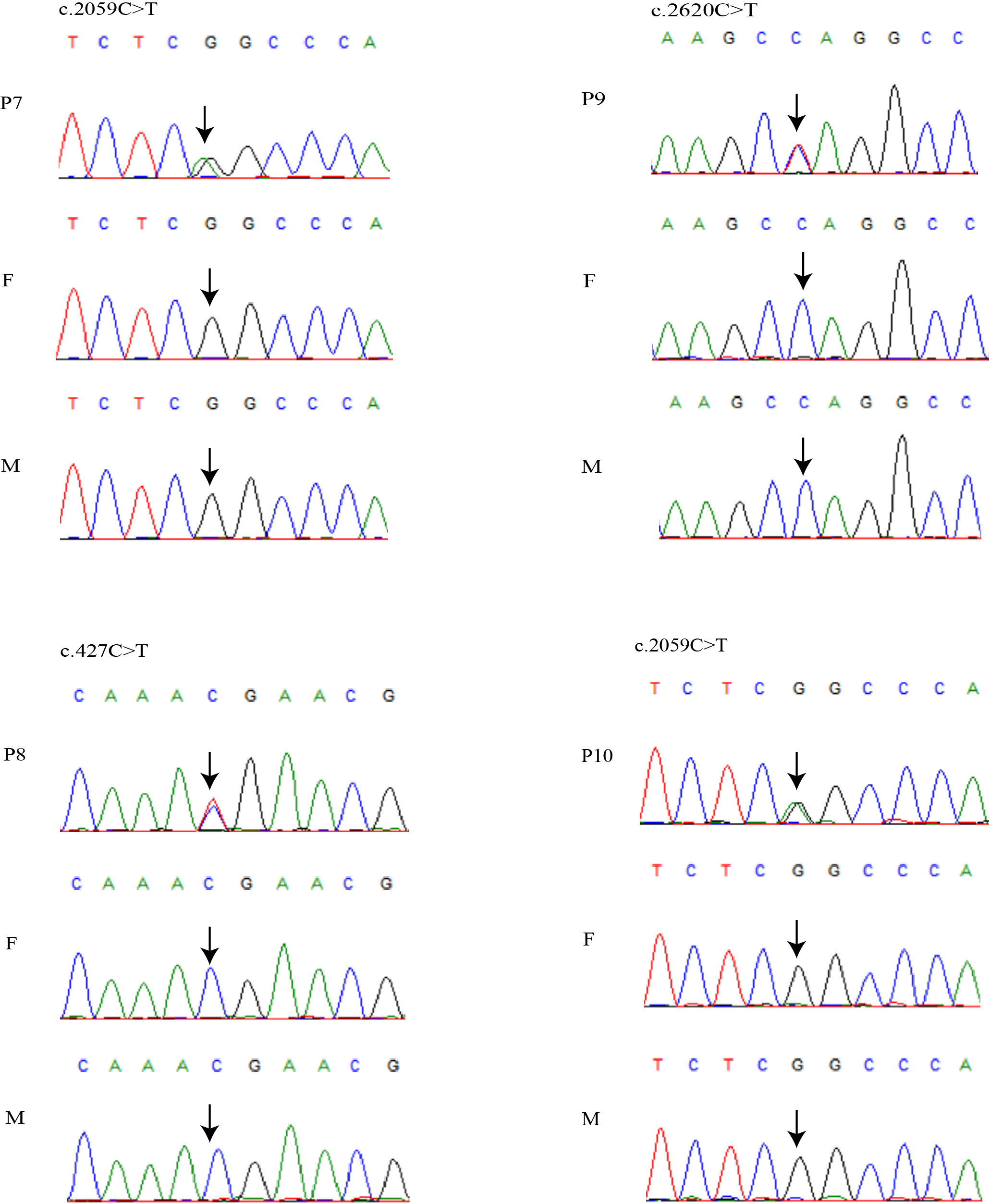

Supplement: Supplementary file 1 [file Image2.TIF]

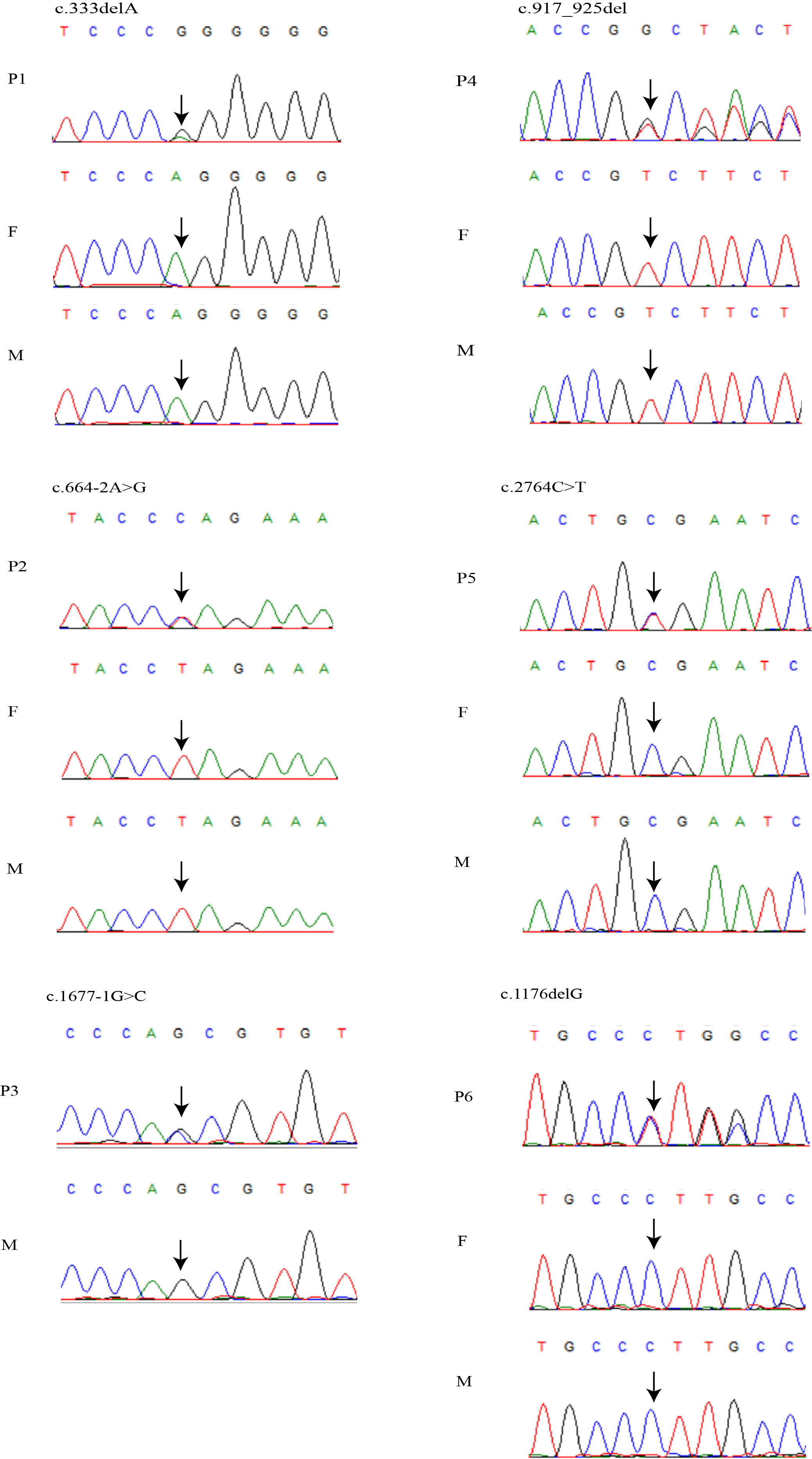

Supplement: Supplementary file 2 [file Image1.TIF]
